# Moral decision-making ‘on the fly’

**DOI:** 10.1007/s00426-025-02126-z

**Published:** 2025-05-08

**Authors:** Petko Kusev, Rose Martin, Paul van Schaik, Joseph Teal

**Affiliations:** 1 LSBU Business School, London South Bank University, London, UK; 2 Department of Psychology, Teesside University, Middlesbrough, UK

## Abstract

Over a century of research has focused on the consistency and inconsistency of human moral decision preferences. We proposed and found that moral decision preferences are flexible and shift towards newly learned moral rules when their application leads to utilitarian choices. Hence, decision-makers’ psychological concept of morality is continually under construction (on the fly); based on learning, informed by changes in moral rules and specific moral contexts. Accordingly, in two experiments we developed and employed a two-stage supervised learning task, where participants learned novel moral rules based on corrective feedback of their moral decisions. Our empirical findings revealed that participants learn new moral rules, transfer these rules to tasks where no feedback is provided. However, participants make decisions based on the principle of maximizing utility rather than a learned rule when the rule conflicts with this principle, demonstrating further the flexibility of moral decision-making. In light of our proposal and findings that decision-makers’ psychological concept of morality is continually under construction, moral decision-making researchers should integrate learning into their respective models and predictions.

**Supplementary Information:**

The online version contains supplementary material available at 10.1007/s00426-025-02126-z.

## Introduction

When making morally sensitive decisions, decision-makers often seek, or *seek to learn,* moral rules to guide their choices. For example, some individuals will pursue consequentialist utilitarian principles which permit that a moral action is one that brings about the best consequence (e.g., sacrificing one life to save the lives of many more; Bentham, 1970; Mill, 2014). Others might adhere to deontological moral rules which explicitly mandate whether or not a particular *action* is morally permissible (e.g., sacrificing a human is always impermissible, even when doing so would save the lives of many more; Kant, 1959). However, evidence from experimental research in psychology reveals that when presented with hypothetical moral decision-making scenarios, people rarely use a consistent set of moral principles to guide their moral behavior. Instead, human behavior often resembles moral flexibility, where preferences for moral choices and principles are flexible and often dependent on the decision-making context. For example, an individual’s preference for a utilitarian choice or judgement can be influenced by numerous contextual factors such as personal involvement (Greene et al., 2001), realism (Bostyn et al., 2018) and accessibility to utilitarian information (Kusev et al., 2016a) in the moral scenario. Moreover, experimental studies which induce learning of moral rules have demonstrated empirically the flexibility of moral preferences. Maier et al. (2023) reveal that humans learn and apply decision strategies (deontological or cost–benefit) based on whether the strategic outcomes were good or bad, indicating flexibility in moral preferences informed by learned decision consequences. Kwon et al. (2022) also reveal that humans demonstrate flexibility in their *application* of moral rules (e.g., queuing behavior), recognizing situations when moral rules can be obeyed and broken. Accordingly, prior research in moral decision-making indicates a consistent pattern; the application of moral principles are flexible, dependent on the context and content of moral dilemmas, and can be learned through experience of ‘good’ and ‘bad’ outcomes.

Whist prior experimental work has explored the learning of novel moral rules associated with existing moral principles (e.g., Nichols et al., 2016), to the best of our knowledge, no previous empirical research has explored moral flexibility for novel rules for which humans have no prior knowledge. In other words, moral rules which are abstract (e.g., based on simple visual cues) and cannot be characterized by established moral principles (e.g., utilitarianism or deontology). In the current project, we accordingly implement a novel supervised learning task in which participants learn abstract moral rules through multiple trials and supervised feedback. We contend that rule-learning is underlying the flexibility of moral preferences. Specifically, human decision-makers seek to learn and indeed succeed in learning novel moral rules which sway moral decisions in the direction of the learned rule. We also propose that when introducing an option which resembles a familiar moral rule (e.g., a utilitarian option), rule learning conflicts with this; rule learning is successful so long as the rule does not conflict with the principle of utility maximization.

### Moral principles and moral flexibility

Traditionally, theorists have considered normative utilitarian decision-making the gold standard for rational moral behavior (e.g., Bentham, 1970; Mill, 2014; von Neumann & Morgenstern, 1947). For example, in trolley dilemmas (Thomson, 1985) and their variations, sacrificing one person to save five is considered rational as it follows the normative utilitarian strategy of maximization. Moreover, moral flexibility is often also considered a human decision-making error; for example, the switch between utilitarian and non-utilitarian moral behavior indicates utilitarian inconsistency (e.g., Greene et al., 2001; Thomson, 1985). However, whilst moral flexibility may disrupt expected normative behavior, it is not necessarily an erroneous decision-making strategy. Recent empirical evidence from behavioral science research indicates that human decision preferences are constructed ‘on the fly’, influenced by the decision-making method of preference elicitation (e.g., Kusev et al., 2020; Pedroni et al., 2017) and feedback (e.g., Kusev et al., 2022). We argue that decision-makers’ psychological concept of morality is continually under construction (on the fly), adapting to environmental feedback such changes in moral rules.

Whilst utilitarian choices are considered rational in some contexts, there exists experimental evidence that deontological moral behavior may have an evolutionary benefit for attracting mates or demonstrating trust and prosocial behavior (Brown & Sacco, 2019; Capraro et al., 2018; Lee et al., 2018; Sacco et al., 2017). Therefore, it would be beneficial and competitively advantageous to switch to deontological principles or acquire entirely new moral rules based on the demands of the (ever-changing) environmental context (Erlandsson, 2021). For example, empirical work on moral economic games reveals that people tend to choose options that are framed as moral as opposed to adopting a consistent strategy (Capraro & Rand, 2018). To put this into a real-world context, historically, people’s moral judgments regarding many aspects of human rights have shifted such as women’s social status, right to vote and the legal recognition and acceptability of same-sex partnerships. Therefore, it is evident that individuals learn and adopt new moral rules and principles that are compatible with general societal norms and laws (FeldmanHall et al., 2018; Lindström et al., 2018), indicating flexibility in accordance with them.

### The acquisition of moral rules and values via learning

Many of the established theories of moral learning have been proposed from a developmental perspective, indicating that our moral values are learned throughout childhood and adolescence. For example, Kohlberg and Hersh’s (1977) empirical work indicates that children right through to adulthood acquire moral understanding through developmental stages. Universal moral grammar theory contends that we are born with an innate moral system, enabling us to acquire complex and universal moral understanding (Mikhail, 2007). More recently, Nichols (2021) emphasizes the capacity for rational improvement through statistical reasoning. In particular, children learn novel moral rules by observing the actions and consequences of other agents, updating their beliefs about the scope of moral rules based on the available evidence of rule application. As accounted for by these theories, moral learning is not limited to child development, but extends into adulthood, enabling the acquisition of new moral rules.

One way in which moral learning can be studied is by exploring the adoption of moral rules via reinforced feedback. Psychological studies in reinforcement learning reveal that over repeated trials human agents can learn to associate stimuli (Wills, 2005). In particular, the frequency with which a human agent demonstrates a particular behavior is determined by the consequences of the behavior; positive and negative reinforcement increases the frequency of the behavior, whilst positive and negative punishment decreases the frequency of the behavior (the predictiveness principle; see Griffiths et al., 2011). Recently, there has been some progress in research on moral learning as a flexible psychological mechanism of moral choice (Crockett, 2013; Cushman et al., 2017). For instance, theoretical models suggest that some of our moral decisions are guided and informed by reinforcement learning systems (Crockett, 2013; Cushman, 2013; Feldmanhall & Dunsmoor, 2019). Specifically, Crockett (2013) proposed that moral learning is guided by past experiences; people are more likely to repeat actions that have produced desirable outcomes in the past, as opposed to actions that have produced undesirable outcomes.

Accordingly, in this article, we build on this work and claim that decision-makers use a simple moral strategy that occurs *on the fly* (continually under construction) and that it is flexible learning that informs moral behavior. Specifically, we contend that morality is learned and updated based on the demands or *rules* set by the context. Therefore, we argue that moral choice is based on the most recently supervised or unsupervised learned successful strategy in that given context when none of the options is dominant in terms of utility.

Whilst the focal point of our work is moral learning of rules through supervised reinforcement learning, we acknowledge the importance of higher-order learning of abstract moral beliefs, values and theories which are also instrumental in guiding moral reasoning and choice. For instance, Oktar et al. (2023) demonstrate how participants’ learning through an introductory moral philosophy course can change their moral views via a reduction in intuition and increased reliance on deliberation. Hence, educational programs can support people to think critically about the evidence regarding opposing views in controversial moral dilemmas. Moreover, we recognize both kinds of moral learning are jointly compatible; contemporary models of reinforcement learning show how learners generate abstract generalizations of rules and the application of these may be dependent on experience and education (Kim & Loewenstein, 2021; Maier et al., 2023). However, to maintain control in exploring the flexibility of moral learning, we have limited our focus to the acquisition and transfer of moral rules by means of a supervised learning task.

### The present experiments

To explore and measure moral rule-learning in adult humans, we conducted two experiments, in which we devised a supervised learning task based on hypothetical moral scenarios. Typically, in supervised learning tasks (commonly used in category learning research in both human and machine learning; e.g., Love et al., 2004; Pothos et al., 2011), the experimental procedure consists of multiple learning trials where decision agents receive corrective feedback on each trial. Supervised learning is often contrasted with unsupervised learning, in which learners are not guided by rules indicated by corrective feedback but instead learn by adopting rules or ‘good’ and ‘bad’ categories spontaneously and without instruction (Love, 2002). Therefore, unlike unsupervised methods, supervised learning requires learners to actively search for rules based on corrective feedback (Love, 2002). New rules acquired from the supervised and unsupervised learning task are supposed to guide and inform agents’ future decisions.

**Fig. 1:**
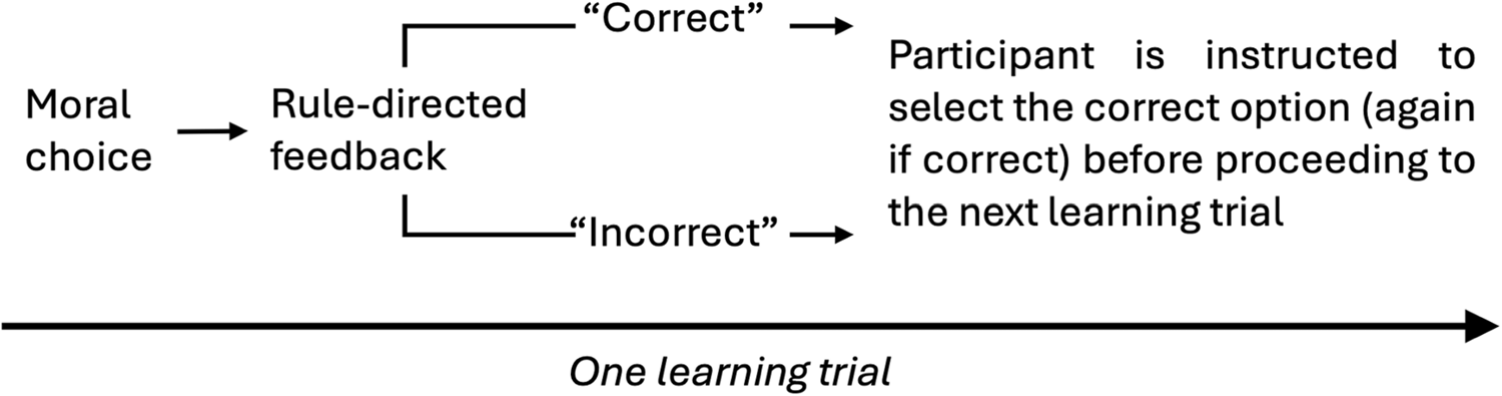
The supervised learning procedure employed in experiments 1 and 2. Note. The unsupervised learning procedure required participants to undertake the same decision task but without corrective feedback

In the present experiments, participants in the experimental conditions learned one of two moral rules which was defined by the characteristics of the visual stimuli that was presented to them on each learning trial. Each trial required participants to select from one of two binary choices and participants received rule-directed feedback after each choice made (see Fig. 1). Accordingly, learning occurred through associations made between the selection of a moral option and corrective choice feedback. In the control group, participants undertook the same choice task, however they did not receive corrective feedback on any of the trials.

We found that participants learn and transfer moral rules to the test phase: a task, where feedback is no longer provided (Experiment 1), and when there is a congruent utilitarian option is available in the test phase of the experiment. In other words, when the learned rule is congruent with the utilitarian option in the test phase, participants demonstrate a preference for this rule. However, when the learned rule is incongruent with the utilitarian option in the test phase, participants prefer the utilitarian option, indicating flexibility in their moral preferences (Experiment 2).

To summarize our argument, we propose the following hypotheses:

- *H1*: Moral decision-making rules acquired through both supervised and unsupervised learning transfer to subsequent unsupervised decision-making when the utility of the options in the environment (test phase) does not change and does not favor either of the options.
- *H2*: Decision-making based on supervised and unsupervised rule-learning alters in response to changes in the utility of decision options in the environment towards more utilitarian choice.

## Experiment 1: moral rule-learning and transfer

The aim of Experiment 1 was to test *H1* by establishing whether participants can (a) learn novel moral rules from a supervised or an unsupervised learning task (learning phase), and (b) transfer learned rules to a test phase where no feedback is provided, and the utility of the choice options remains equal.

### Method

#### Participants

One hundred and thirty participants (63 female) were recruited via an online recruitment service and rewarded for their participation. Their mean age was 53 (*SD* = 13.74). Research ethics approval was obtained before data collection. All participants provided their informed consent prior to taking part in the study and were treated in accordance with the British Psychological Society’s and American Psychological Association’s codes of ethics and conduct.

For statistical testing in both experiments, we set a significance level of 0.01. We did not assume an effect size, but wanted to ensure that our sample size would allow us to detect a large effect size (*d* = 0.8, by convention; Cohen, 1988). We ran the experiment for 14 days to ensure data collection from a sufficiently large sample for a large effect size to achieve a statistical power of at least 0.80. According to retrospective power analysis, the achieved sample size (*N* = 130) produced a power of at least 0.86 for each of the *t*-tests in Experiment 1, which was sufficient to achieve our target.^1^

All data and R codes for Experiments 1 and 2 are available at OSF: https://osf.io/qj4dc/?view_only=4bb7bef36917411294c5bbc81315a80c. The two experiments (studies and analyses) reported in this article were not pre-registered.

#### Experimental design

The experiment used a one-factor between-subjects design. The single independent variable was rule-learning, with three levels. There were two experimental conditions (supervised rule 1-learning, and supervised rule 2-learning) and one control condition (unsupervised rule-learning). In the supervised rule 1-learning (moral rule 1) and supervised rule 2-learning (moral rule 2) conditions participants received feedback about the correctness of their choices according to moral rules. The moral rules were determined by the characteristics of the two choice options which were represented by visual stimuli. Accordingly, participants learned to associate specific characteristics of the choice options with the terms ‘correct’ or ‘incorrect’. What constituted correct or incorrect responses depended on the experimental condition the participants were allocated to (i.e., supervised rule 1-learning, and supervised-rule 2 learning). In the control condition (unsupervised rule-learning), participants received no feedback regarding their choices. First, we measured the influence of rule-learning on learning consistency (proportion of decisions employing the learned rule in the learning phase of the experiment), and, second, we tested transfer consistency (proportion of decisions employing the learned rule in the test phase of the experiment) from the learning phase to the test phase. It is important to note here that learning consistency indicates how successfully a rule is being learned, in each of the two experimental conditions. Likewise, transfer consistency is used to ascertain how successfully a rule is being transferred from the learning phase to the test phase of the experiment (where no feedback is provided) in each of the two experimental conditions. For example, learning consistency under the supervised rule 1-learning condition was calculated as the proportion of trials in which a participant responded with a decision that was consistent with Rule 1 in the learning phase. Transfer consistency was calculated the same way for responses in the test phase. Learning consistency and transfer consistency under the supervised rule 2-learning condition were calculated the same way, but with rule 2 substituted for rule 1. In the learning phase and test phase of the control condition, the proportion of decisions consistent with moral rule 1 was calculated, as was the proportion of decisions consistent with moral rule 2. The proportions from the learning phase and test phase were then used to analyze transfer consistency, as explained in the Transfer Consistency section of Experiment 1 results.

#### Materials and procedure

An interactive online computer-based task for binary decision-making was used. The task required all participants to complete a 2-stage experiment which involved a learning phase (30 randomized trials), and a test phase (20 randomized trials) immediately after. In each trial of the learning phase, participants were presented with a moral dilemma and task instructions. Hence, the moral scenario provided participants with the moral context for which they were making their moral choices:

Two groups of strangers – Group A and Group B – have been taken hostage by armed kidnappers. The kidnappers intended to kill both groups; however, they have given you the chance to save one of the groups. *The group that you decide to save will be released unharmed by the kidnappers. The group that you decide not to save will be killed by the kidnappers.* Please indicate which group you decide to **save** by clicking on the chosen group.

**Fig. 2:**
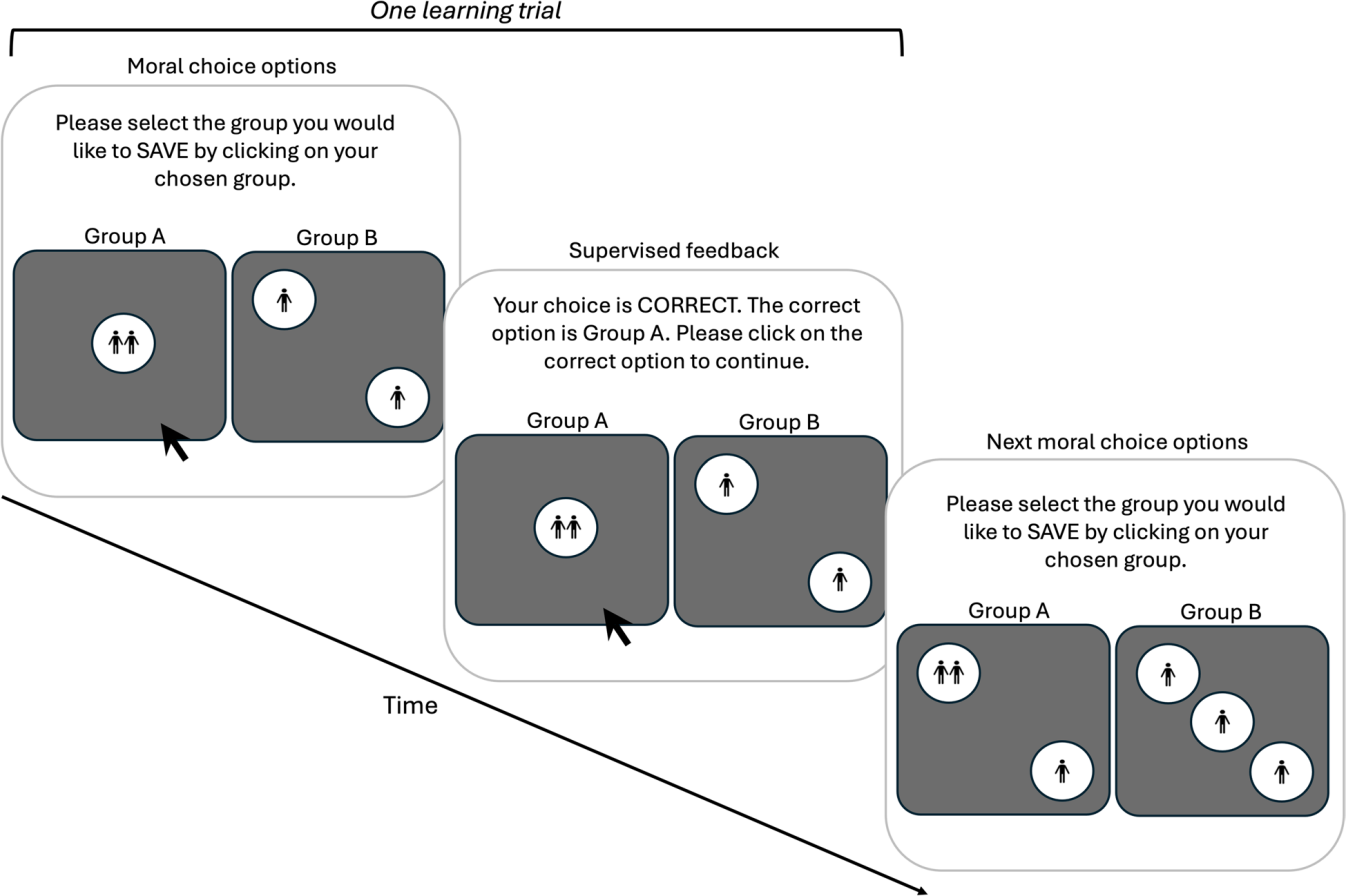
The reinforcement learning procedure employed in experiments 1 and 2. Note. An example of the learning phase under moral rule 1 (the correct answer is the option with the smaller number of pips)

Groups A and B were represented by visual stimuli which resembled 2D images of a pair of blue dice (one die for each group), where only one face of each die was visible. Silhouettes of people were presented inside the die’s circles (pips). These silhouettes represented utility: the number of people that could be saved (see Fig. 2 for an example and Table S1 and S2 in the supplementary material, for details on all trial-by-trial stimuli).

Whilst the moral scenario description may have been familiar to participants, we intentionally designed stimuli and rules that we assumed participants had no prior experience with. In all trials, the number of people in each of the two choice options was equal, and thus a utilitarian strategy could not be applied by participants. However, the number of pips on the dice and the number of people per pip differed between the two dice. Specifically, Moral Rule 1, used in experimental condition supervised rule 1-learning, provided the feedback ‘correct’ after participants chose the group with the largest number of people in a pip (thus, it was the option with fewer pips). By contrast, moral rule 2, which was used in experimental condition supervised rule 2-learning, provided the feedback ‘correct’ after participants choose the group with the largest number of pips (see Fig. 2).

Participants received feedback after every choice made, indicating whether they were correct or incorrect with regards to their choice (unless they were in the unsupervised-rule-learning condition where no feedback was provided). After receiving feedback based on their choice, participants learning under moral rule 1 and moral rule 2 were further prompted to select the correct option (even if their choice was correct) for the same choice problem (see Fig. 2). In the control condition, no feedback was given, but after making their choice, participants had to confirm this choice.

In the test phase of all conditions, participants were presented with a further 20 trials of a similar task (same dilemma as in the learning phase, but different choice pairs); however, no feedback was presented to the participants. The role of the test phase of the experiment was to measure the transfer of learning to a task where feedback was no longer provided to guide participants’ choices.

### Results and discussion

#### Learning consistency

**Fig. 3:**
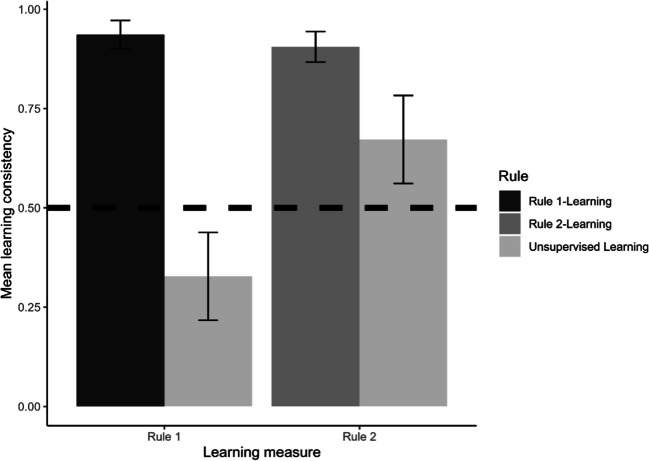
Learning consistency as a function of study condition. Note. Error bars represent 99% confidence intervals of the means

Welch *t*-tests and *t*-tests against a constant were conducted to investigate the influence of supervised and unsupervised rule-learning on learning consistency. The results, presented in Table 1 and Fig. 3, revealed that the rule-directed supervised feedback of moral rule 1 (supervised rule 1-learning condition) induced higher learning consistency with moral rule 1 than when no feedback (unsupervised rule-learning condition) was provided. Similarly, the rule-directed supervised feedback of moral rule 2 (supervised rule 2-learning condition) induced higher learning consistency with moral rule 2 than when no feedback (unsupervised rule-learning condition) was provided. Learning consistency did not differ significantly between the two experimental supervised learning conditions. The results also revealed that the rule-directed supervised feedback of moral rule 1 (supervised rule 1-learning condition) induced higher learning consistency than chance. The same was true for the rule-directed supervised feedback of moral rule 2 (supervised rule 2-learning condition). Moreover, decision-making was more consistent with moral rule 2 in the unsupervised rule-learning condition.

**Table 1:** Statistical comparisons for learning consistency (Experiment 1)

Comparison	t	df	p	d	95% Cl(d)	
					Lower limit	Upper limit
Rule 1-learning – unsupervised learning	20.56	51.88	< .001	2.25	1.54	2.95
Rule 2-learning–unsupervised leaming	4.01	53.15	< .001	0.85	0.27	1.43
Rule 1-learning – Rule 2-learning	1.18	83.21	.240	0.26	−0.30	0.81
Rule 1-learning – chance (0.50-0.50)	24.68	43.00	< .001	3.72	2.64	4.84
Rule 2-learning – chance (0.50-0.50)	21.32	41.00	< .001	3.29	2.29	4.31
Unsupervised learning – chance (0.50-0.50)	3.14	43.00	.003	0.47	0.06	0.88

In sum, preferences for moral rule 1 and moral rule 2 were learned under supervised reinforcement learning to a high degree. Under unsupervised learning, preferences for moral rule 2 were also learned, but to a lesser degree. However, this advantage (preference for moral rule 2) did not further enhance the supervised learning of Moral Rule 2: Rule 2 was not learned better than moral rule 1 under supervised learning.

#### Transfer consistency

We used a plan for transfer analysis (see Table 2) to guide the analysis of transfer consistency; specifically, we analyzed whether participants transferred the moral rule learned in the learning phase to the test phase of the experiment, in terms of maintaining or increasing the rate of decisions consistent with the learned rule. Note that learning (and, therefore, transfer) differs conceptually and qualitatively between supervised and unsupervised learning: in supervised learning, normative feedback occurs to regulate empirically participants’ decision-making on each trial, while in unsupervised learning, decision-making is not regulated. Because of this conceptual and qualitative difference, we analyze transfer after supervised learning differently from transfer after unsupervised learning: in supervised learning, transfer in the test phase is evaluated in relation to a normative response that was learned in the learning phase. Specifically, in the supervised rule-learning conditions of Experiment 1, the dependent variable in transfer analysis is the rate of decisions in the test phase according to the rule that is learned in the learning phase (in relation to the rate achieved in the learning phase): rule 1-decisions for moral rule 1-supervised learned and rule 2-decisions for moral rule 2-supervised learning (see Table 2).

**Table 2:** Analysis of transfer (Experiment 1)

Learning phase	Test phase	Dependent variable	Congruence of learning-and test phase	Expected transfer	*t*	*df*	*p*	*d*	CI, LL	CI, UL
Supervised, Rule 1	No utility-dominant rule	Rule 1-decisions	Not applicable	Positive	2.52	43	.02	0.38	−0.02	0.78
Supervised, Rule 2	No utility-dominant rule	Rule 2-decisions	Not applicable	Positive	0.54	41	.54	0.08	−0.32	0.48
Unsupervised	No utility-dominant rule	Rule 2-decisions	Not applicable	Positive	0.05	43	.96	0.01	−0.38	0.40

CI, LL: lower limit of 99% confidence interval. CI, UL: upper limit of 99% confidence interval

However, in unsupervised learning, transfer is evaluated in relation to the dominant response that was measured in the learning phase. The dependent variable is the rate of decisions in the test phase for the option that was dominant in the learning phase: decisions consistent with Rule 2, according to our results from the learning phase of Experiment 1 (mean = 0.67).

**Fig. 4:**
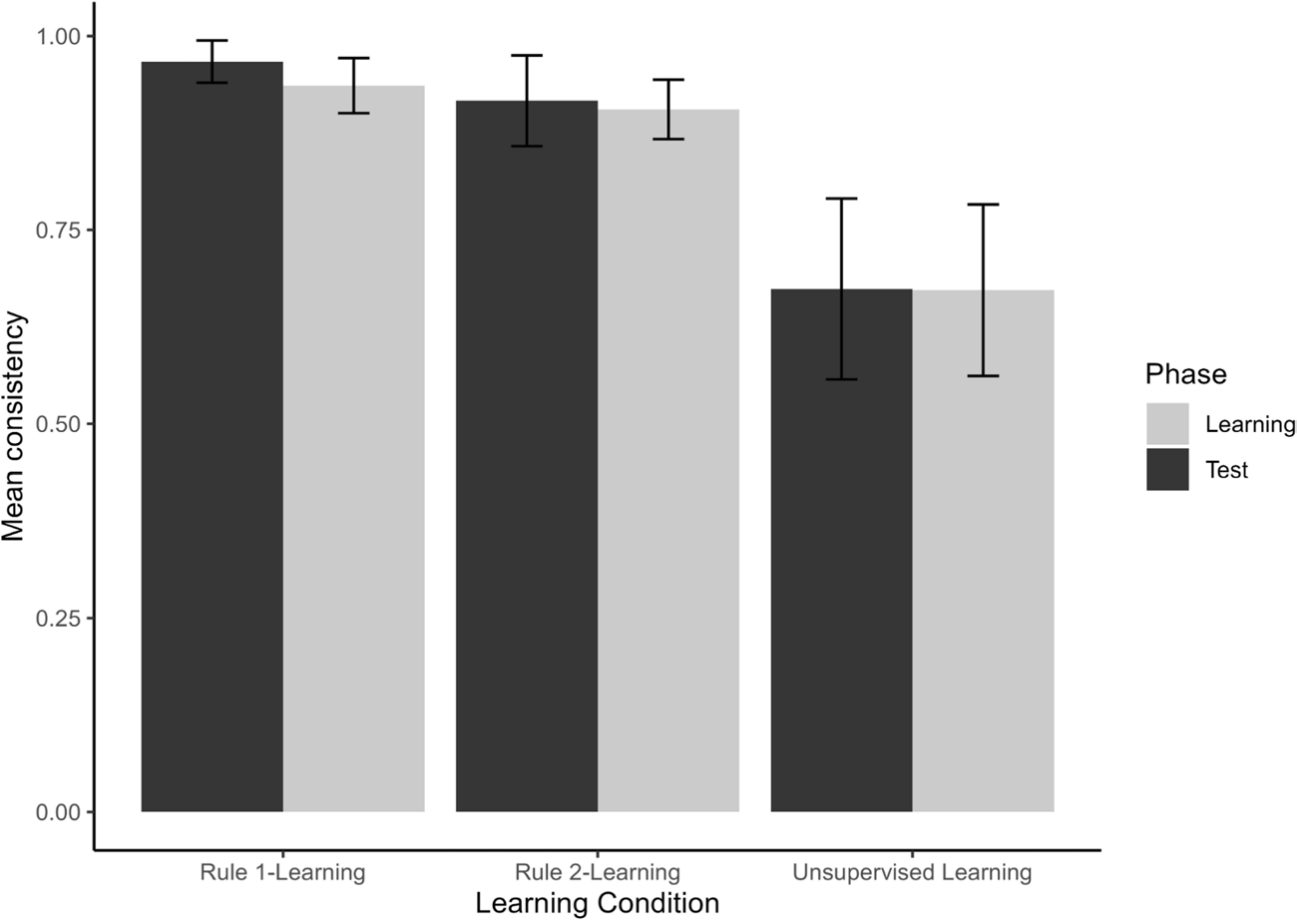
Transfer consistency: change from learning phase to test phase as a function of study condition. Note. Error bars represent 99 %-confidence intervals of the means

We expect positive transfer (maintaining or increasing the rate of the normative or dominant decision from the learning phase) in all three conditions, as there was no manipulation to counter the learning that had occurred in the learning phase. Required evidence for positive transfer is a statistically significant positive change or a non-significant change; evidence against is a statistically significant negative change. The results (see Fig. 4 and Table 2) show evidence for positive transfer in both supervised and unsupervised learning, with a non-significant positive change each time. The size of the non-significant effect was middling under supervised moral rule 1-learning, and small or negligible for supervised moral rule 2-learning and unsupervised learning. In sum, the results provide evidence for transfer of learning under both supervised reinforcement learning and unsupervised learning conditions.

#### Psychological mechanism of moral learning

Furthermore, the results revealed that the associations between learning consistency and transfer consistency were positive, strong, and statistically significant for (a) moral rule 1-learning, *r* (43) = 0.72, *p* <. 001, (b) moral rule 2-learning, *r* (41) = 0.70, *p* < .001, and (c) unsupervised rule-learning*, r* (43) = 0.88, *p* < .001. The significant correlations between learning consistency and transfer consistency for both moral rule 1-learning and moral rule 2-learning further indicate that participants’ learned moral-decision preferences were subsequently used in the test phase of the experiment where feedback was no longer provided to guide participants’ decisions. The significant correlation between learning consistency and transfer consistency with unsupervised rule-learning suggests that participants adopted a decision strategy of their own (a preference for a rule) and continued to employ it throughout the trials.

To provide further evidence for these conjectures supported by these correlational results, mediation analysis was conducted. The R package *lavaan* (Rosseel, 2012) was used to test whether learning consistency mediates the effect of rule-directed supervised feedback on transfer consistency. According to our mediation model, rule-learning (supervised or unsupervised; as an independent variable) is the determinant of learning consistency and, in turn, this consistency (as a mediator) is the determinant of transfer consistency (as the dependent variable). According to modern mediation analysis (Hayes, 2022), the total effect of the independent variable on the dependent variable is broken down into two effects. First, the direct effect is that part of the effect of the independent variable, which does not overlap with the mediator. Second, the indirect effect is the multiplication of the effect of the independent variable (feedback) on the mediator with the effect of the mediator (learning consistency) on the dependent variable (transfer consistency). We tested for significance with bootstrapping (*N* = 5000) because the indirect effect is commonly not normally distributed. The results showed full mediation of the effect of rule-directed supervised feedback on transfer consistency by learning consistency (Table 2). Panel A, Table 3 shows the results of an analysis where the levels of rule-learning with feedback are supervised rule 1-learning and unsupervised learning. Rule 1 was a significant positive predictor of learning consistency; learning consistency, in turn, was a significant predictor of transfer consistency. The direct effect of Rule 1 on transfer consistency was not significant, but the indirect effect was. Panel B, Table 3 shows the results of analysis where the levels of rule-learning with feedback are supervised rule 2-learning and unsupervised learning. The pattern of results is the same as in Panel A, but now for rule 2 instead of rule 1. In sum, these results provide evidence that the effect of rule-learning (supervised or unsupervised; as an independent variable) in the learning phase on transfer consistency in the test phase, was explained by its effect on learning consistency in the learning phase as a psychological mechanism of moral learning.

**Table 3:** Mediation analyses (Experiment 1)

Antecedent	Consequent	beta	SE	*z*	*p*	99%-lower limit	99%-upper limit
Rule 1	Learning cons	0.75	0.05	14.73	< .001	0.65	0.85
Learning cons	Transfer cons	0.85	0.07	12.29	< .001	0.72	0.99
Rule 1 (direct)	Transfer cons	0.12	0.09	1.36	.173	−0.05	0.28
Rule 1 (indirect)	Transfer cons	0.64	0.08	8.21	< .001	0.49	0.80
Panel B: Moral Rule 2-learning versus no moral-rule-learning							
Antecedent	Consequent	beta	SE	*z*	*p*	99%-lower limit	99%-upper limit
Rule 2	Learning cons	0.39	0.08	5.22	< .001	0.25	0.54
Learning cons	Transfer cons	0.86	0.03	25.66	< .001	0.79	0.92
Rule 2 (direct)	Transfer cons	0.04	0.05	0.74	.461	−0.06	0.13
Rule 2 (indirect)	Transfer cons	0.34	0.06	5.23	< .001	0.21	0.46

Values for the effects are standardized. Consistency is denoted by ‘cons’

## Experiment 2: learning moral rules and expected moral utilitarian behavior

Having established in Experiment 1 that moral rules can be both learned and transferred to situations where no feedback is provided, the aim of Experiment 2 was to test Hypothesis *H2* by investigating whether decision-making based on supervised and unsupervised rule-learning alters in response to changes in the utility of decision options in the environment (Experiment 2). Accordingly, the purpose of Experiment 2 was to investigate whether learned moral rules are informed by the newly acquired utilitarian information in the test phase.

### Method

#### Participants

Two hundred and forty participants (118 female) who had not taken part in Experiment 1 were recruited via an online recruitment service and rewarded for their participation (£1). Their mean age of the participants was 52 (*SD* = 13.65). Ethical approval and informed consent was obtained before data collection and all participants were treated in accordance with the British Psychological Society’s and American Psychological Association’s codes of ethics and conduct.

We did not assume an effect size, but wanted to ensure that our sample size would allow us to detect a large effect size in *t*-tests (*d* = 0.80 by convention; Cohen, 1988) and ANOVA (*f* = 0.40 by convention; Cohen, 1988). We ran the experiment for 14 days to ensure data collection from a sufficiently large sample for a large effect size to achieve a statistical power of at least 0.80. According to retrospective power analysis, the achieved sample size (*N* = 240) produced a power of at least 0.88 for each of the *t*-tests and ANOVAs, which was sufficient to achieve our target.^2^

#### Experimental design

A 3 × 2 independent measures design was employed in a computer-based experiment. Accordingly, there were six experimental conditions. The first independent variable (rule-learning) was identical to the independent variable in Experiment 1. The second independent variable was utilitarian congruence, with two levels: whether the learned rule became the utilitarian option in the test phase (congruent) or not (incongruent). For example, when the learned rule was not the utilitarian option in the test phase, participants were required to choose between the option that was consistent with the learned rule or the option that was consistent with utilitarian choice (the option that allowed them to maximize utility). For Experiment 2, we measured learning consistency (in the learning phase) and transfer consistency (in the test phase) as in Experiment 1, and utilitarian choice (moral decision preferences based on maximization of utility in the test phase).

#### Materials and procedure

The participants in Experiment 2 were presented with the same 30 randomized learning trials as were used in Experiment 1 and then 20 new randomized test trials, all of which contained the same dilemma description as Experiment 1. Crucially, as in Experiment 1, the learning phase contained choice options with equal utility, but the test phase contained options with unequal utility; a utility-dominant option was now available (see Table S2, supplementary material for details on all trial-by-trial stimuli).

### Results and discussion

#### Learning consistency

**Fig. 5:**
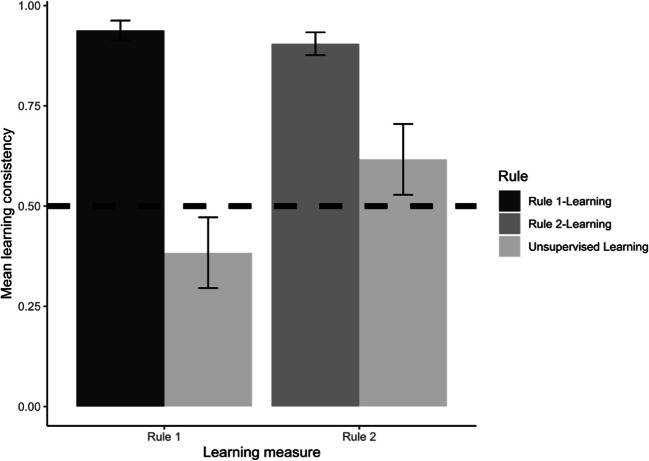
Learning consistency as a function of study condition (Experiment 2). Note. Error bars represent 99% confidence intervals of the means

The same analysis was conducted as in Experiment 1 to analyze learning consistency. The results presented in Table 4 and Fig. 5, in comparison with those in Table 1 and Fig. 3, demonstrate that the pattern of results for learning consistency was the same in both experiments. Therefore, in both experiments preferences for moral rule 1 and moral rule 2 were learned under supervised reinforcement learning to a high degree. Under unsupervised learning, preferences for moral rule 2 were also learned, but to a lesser degree.

**Table 4:** Statistical comparisons for learning consistency (Experiment 2)

Comparison	*t*	*df*		*p*	*d*	*95% CI(d)*	
						Lower limit	Upper limit
Rule 1-learning – unsupervised learning	12.03	91.62	<	.001	1.90	1.41	2.39
Rule 2-learning – unsupervised learning	6.19	95.20	<	.001	0.98	0.55	1.41
Rule 2-learning – Rule 2-learning	1.74	155.51		.080	0.27	−0.15	0.68
Rule 1-learning – chance (0.50-0.50)	34.84	79.00	<	.001	3.89	3.06	4.73
Rule 2-learning – chance (0.50-0.50)	28.36	79.00	<	.001	3.17	2.47	3.89
Unsupervised learning – chance (0.50-0.50)	2.62	79.00		.010	0.29	0.00	0.59

In sum, as in Experiment 1, preferences for moral rule 1 and moral rule 2 were learned under supervised reinforcement learning to a high degree. Also as in Experiment 1, despite the advantage of moral rule 2 under unsupervised learning, moral rule 2 was not learned better than moral rule 1 under supervised reinforcement learning.

#### Transfer consistency

In this analysis, the transfer is partially confounded with the utility manipulation. However, it is important to conduct transfer consistency analysis in order to ensure that there are not any unexpected (e.g., different from Experiment 1) shifts in participants’ decision-making. As in Experiment 1, the analysis of transfer consistency was guided by the plan for transfer analysis (see Table 5). As we argued in Experiment 1, learning (and, therefore, transfer) differs conceptually and qualitatively between supervised and unsupervised learning. Therefore, we analyze transfer after supervised learning differently from transfer after unsupervised learning. Accordingly, we used the same dependent variables as in Experiment 1 (see Table 5).

**Table 5:** Analysis of transfer (Experiment 2)

Learning phase	Test phase	Dependent variable	Congruence of learning-and test phase	Expected transfer	*t*	*df*	*p*	*d*	CI, LL	CI, UL
Supervised, Rule 1	Rule 1 utility-dominant	Rule 1-decisions	Congruent	Positive	1.56	39	.13	0.25	−0.17	0.66
Supervised, Rule 1	Rule 2 utility-dominant	Rule 1-decisions	Incongruent	Opposite	−8.70	39	< .001	−1.38	−1.95	−0.80
Supervised, Rule 2	Rule 2 utility-dominant	Rule 2-decisions	Congruent	Positive	3.74	39	.001	0.59	0.15	1.03
Supervised, Rule 2	Rule 1 utility-dominant	Rule 2-decisions	Incongruent	Opposite	−6.84	39	< .001	−1.08	−1.59	−0.56
Unsupervised	Rule 2 utility-dominant	Rule 2-decisions	'Congruent'	Positive	2.99	39	.005	0.47	0.04	1.06
Unsupervised	Rule 1 utility-dominant	Rule 2-decisions	'Incongruent'	Opposite	−6.73	39	< 0.001	−1.06	−1.57	−0.55

CI, LL: lower limit of 99% confidence interval. CI, UL: upper limit of 99% confidence interval

Positive transfer is expected in the congruent supervised rule-learning conditions, as the manipulation of the utility-dominant option will nudge decision-making in the direction of the normative response from the learning phase of the study. Moreover, positive transfer is also expected in the ‘congruent’ unsupervised rule-learning condition (presentation that is congruent with the dominant response from the learning phase). Accordingly, we expect a positive transfer because of congruence between the learned (normative or dominant) responses from the learning phase and the utility-dominant option from the test phase of the study. Therefore, the evidence required for positive transfer is a statistically significant positive change or a non-significant change.

**Fig. 6:**
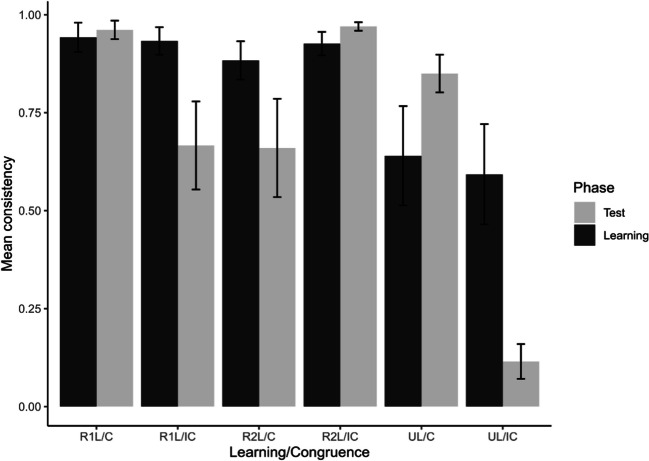
Transfer consistency: change from learning phase to test phase as a function of study condition (Experiment 2). Note. Error bars represent 99% confidence intervals of the means

The results (see Fig. 6 and Table 5) show evidence for positive transfer in both supervised learning in the congruent conditions in the test phase, and in the ‘congruent’ unsupervised rule-learning condition. In the congruent rule 2 condition and the ‘congruent’ unsupervised condition, the positive change with a middling effect size was significant towards the normative response or the dominant response from the learning phase. In the congruent rule 1 condition, the small effect size in the same positive change direction was not significant.

Negative transfer is expected in the incongruent supervised rule-learning conditions, as the manipulation of the utility-dominant option will nudge decision-making in the opposite direction of the normative response from the learning phase of the study. Moreover, negative transfer is also expected in the ‘incongruent’ unsupervised rule-learning condition (presentation that is incongruent with the dominant response from the learning phase). This occurs where the utility-dominant option followed rule 1, as this manipulation will nudge decision-making in the direction of rule 1, while in the learning phase rule 2-consistent decisions were dominant. Accordingly, we expect a negative transfer because of incongruence between the utility-dominant option in the test phase and the dominant response from the learning phase of the study. Therefore, the evidence required for negative transfer is a statistically significant negative change.

The results (see Fig. 6 and Table 5) show evidence for negative transfer, with statistically significant effects and large effect sizes, in the direction opposite of the normative response or dominant response from the learning phase. These results occurred in both the supervised rule-learning and the unsupervised rule-learning conditions.

#### Flexibility analysis

Building on the analyses of transfer in Experiments 1 and 2, we carried out flexibility analyses to provide evidence to support or refute our claim of flexibility of moral decision-making. An analysis of the effect sizes from the two experiments collectively was conducted to estimate the flexibility in moral decision-making. Specifically, we analyzed to what extent the original effect size of the transfer (estimated as *d* from Experiment 1) can be increased or reversed by presenting a utility-dominant option in the test phase to achieve an enhanced effect (estimated as *d* from Experiment 2).

*Increasing positive transfer with an utility-dominant option available in the test phase of the study:* according to our *flexibility analysis*, from the congruent rule 2-learning condition and the ‘congruent’ unsupervised rule-learning condition the estimated ‘benefit’ in the direction of the learned rule in the learning phase was approximately half a standard deviation. Specifically, from the results of Table 2, (Experiment 1) and Table 5 (Experiment 2), for the congruent rule 2-learning condition, the increase in *d* was 0.59—0.08 = 0.51 and for the ‘congruent’ unsupervised rule-learning condition, the increase in *d* was 0.47—0.01 = 0.46.

From the congruent rule 1 condition the estimated ‘loss’ was less than this benefit. The decrease in *d* was 0.25—0.38 = −0.13, when the original effect size was middling (*d* = 0.38). This result may indicate a ceiling effect, with the initial (learning-phase) rate of rule-consistent decisions exceedingly high (mean = 0.94 in both Experiment 1 and Experiment 2) that was then increased further in the test phase (mean = 0.97 and 0.96, respectively).

*Inducing negative transfer with an utility-dominant option available in the test phase of the study:* our *flexibility analysis* further showed exceedingly large changes in the direction of the utility-dominant option in the test phase away from the learned response (normative or dominant) in the learning phase. Specifically, from the results of Table 2 (Experiment 1) and Table 5 (Experiment 2), for the incongruent rule 1-learning condition, the decrease in *d* was −1.38—0.38 = −1.76; for the incongruent rule 2-learning condition, the decrease in *d* was −1.08—0.08 = −1.16; for the ‘incongruent’ unsupervised rule-learning condition, the decrease in *d* was –1.06—0.01 = −1.07.

#### Summary of transfer consistency and flexibility

The results show evidence for positive transfer (in the congruent conditions) from the learned normative or dominant responses (in the learning phase of the study) to the responses in the test phase. This happened when the utility-dominant option in the test phase was also congruent with the learned responses from the learning phase. Moreover, the results from the flexibility analysis revealed that with congruent rule-learning conditions the original effect size of positive transfer (Experiment 1) was increased by presenting a utility-dominant option in the test phase of Experiment 2.

Furthermore, the results also demonstrate great flexibility of moral decision-making with negative transfer. For example, in the incongruent conditions participants altered their initially learned responses (consistent with the learned rule) to the utility-dominant option in the test phase of the study. Moreover, the results from the flexibility analysis revealed that with incongruent rule-learning conditions the original effect size of positive transfer (Experiment 1) was reversed to negative by presenting an utility-dominant option in the test phase of Experiment 2. This result indicates induced negative transfer with a utility-dominant option available in the test phase of Experiment 2.

#### Utilitarian choice in the test phase

As the research design included the manipulation of utility of one of the choice options in the test phase, an analysis of this manipulation was undertaken. An initial analysis with related *t*-tests showed that, in all conditions, the rate of decision-making according to the utility-dominant option was above chance (all *p* ≤ .01). Moreover, transfer analysis (presented in the previous section) had shown a significant shift in decision-making towards the utility-dominant option in the incongruent supervised rule-learning conditions and the ‘incongruent’ unsupervised rule-learning conditions in the test phase. Therefore, an effect of utility dominance was established.

**Fig. 7:**
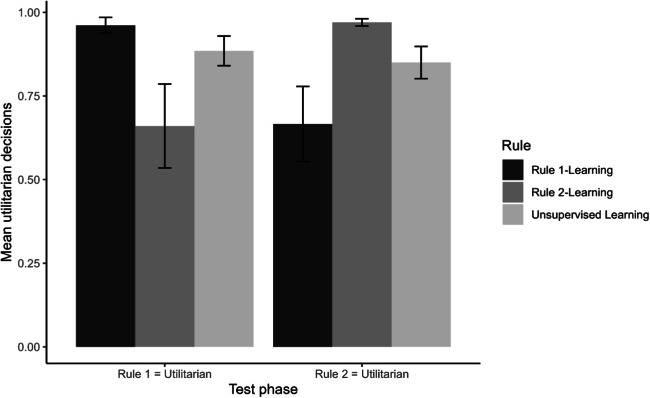
Utilitarian decision-making as a function of study condition (Experiment 2). Note. Error bars represent 99% confidence intervals of the means

However, the pattern of results in the test phase (see Fig. 7) differed between (a) the conditions with the option corresponding with moral rule 1 that was dominant in terms of utility and (b) the conditions with the option corresponding with moral rule 2 that was dominant in terms of utility. Therefore, the two sets of conditions are analyzed separately in the following two subsections.

#### Utilitarian choice under supervised rule-learning 1

One-way ANOVA was conducted to test the effect of rule-learning on utilitarian choice under the conditions where, in the test phase, the utility-dominant option was consistent with moral rule 1. The effect was significant, *F(*2, 117) = 16.48, *p* < 0.001, ${\eta }^{2}$ =0.22. Post hoc tests with Bonferroni correction showed that the difference between the supervised rule 1-learning condition (*M* = 0.96; *SD* = 0.07) and the supervised rule 2-learning condition (*M* = 0.66; *SD* = 0.39) and between the unsupervised rule-learning condition (*M* = 0.89; *SD* = 0.14) and the supervised rule 2-learning condition (both *p* < 0.001) were significant. The difference between the supervised rule 1-learning condition and the unsupervised rule-learning condition (*p* = 0.49) was not significant.

The results also revealed that when the learned moral rule 1 was congruent with the utility-dominant option in the test phase, the association between learning consistency and utilitarian choice preferences in the test phase was positive and statistically significant, *r* (38) = 0.77, *p* < .001. However, crucially, when the learned moral rule 2 was not congruent with the utility-dominant option in the test phase, the association between learning consistency and utilitarian choice preferences in the test phase was negative and statistically significant, *r* (38) = −0.63, *p* < .001. For participants in the unsupervised rule-learning condition, there was no significant relationship between learning consistency and utilitarian choice preferences in the test phase when the option corresponding with moral rule 1 was dominant in terms of utility, *r* (38) = −0.20, *p* = 0.21. As in Experiment 1, the results of the correlational analyses, therefore, suggest further that participants who learned a rule (supervised learning), also used it in the test phase when a utility-dominant option was available. Conversely, participants who learned a rule (supervised learning) that was incongruent with the utility-dominant option in the test phase switched to choosing the utility-dominant option (rule 1) in the test phase. The non-significant negative correlation in the unsupervised rule-learning condition indicates a tendency to switch from the learned rule 2 to the utility-dominant option that corresponds with rule 1 in the test phase.

#### Utilitarian choice under supervised rule 2-learning

One-way ANOVA was conducted to test the effect of rule-directed supervised feedback on utilitarian choice under the conditions where, in the test phase, the utility-dominant option was consistent with moral rule 2. The effect was significant, *F(*2, 117) = 19.11, *p* < .001, ${\eta }^{2}$ = 0.25. Post hoc tests with Bonferroni correction showed that the difference between the supervised rule 2-learning condition (*M* = 0.97; *SD* = 0.03) and the supervised rule 1-learning condition (*M* = 0.67; *SD* = 0.35) and between the unsupervised rule-learning condition (*M* = 0.85; *SD* = 0.15) and the supervised rule 1-learning condition (both *p* < .001) were significant. The difference between the unsupervised rule-learning condition and the supervised rule 2-learning condition (*p* = .05) was not significant.

Furthermore, the results revealed that when the learned moral rule 2 was congruent with the utility-dominant option in the test phase, the association between learning consistency and utilitarian choice preferences in the test phase was positive and statistically significant, *r* (38) = 0.70, *p* < .001. However, crucially, when the learned moral rule 1 was not congruent with the utility-dominant option in the test phase, the association between learning consistency and utilitarian choice preferences was negative and statistically significant, *r* (38) = −0.71, *p* < .001. For participants in the unsupervised rule-learning condition, there was no significant relationship between learning consistency and utilitarian choice preferences in the test phase when the option corresponding with Moral Rule 2 was dominant in terms of utility, *r* (38) = −0.15, *p* = .37. As in Experiment 1, these results suggest further that participants who learned a rule (supervised learning), also used it in the test phase even when a utility-dominant option was available. Conversely, participants who learned a rule (supervised learning) that was incongruent with the utility-dominant option in the test phase switched to choosing the utility-dominant option (rule 2) in the test phase. The non-significant negative correlation in the unsupervised rule-learning condition indicates a tendency to switch from the learned Rule 1 to the utility-dominant option that corresponds with rule 2 in the test phase.

#### Summary of utilitarian choice

The results of utilitarian choice in the test phase show a positive effect of utility dominance. However, the extent of the effect varied. First, under supervised reinforcement learning, congruence of learning-phase and test-phase results in more utilitarian decision-making than incongruence. Second, unsupervised learning, whether ‘congruent’ or ‘incongruent’, also results in more utilitarian decision-making. Finally, supervised rule-learning conditions and unsupervised rule-learning conditions with the same utility-dominant option in the test phase do not differ in utilitarian decision-making. Therefore, unsupervised learning is most flexible in the sense that with a utility-dominant option in the test phase, utilitarian decision-making can match that of supervised learning under congruence and exceed that of supervised learning under incongruence. The correlational results in Experiment 2 suggest further support for the psychological mechanism of moral learning that was established in Experiment 1. The positive correlations between learning consistency and transfer consistency in the congruent supervised rule-learning conditions suggest that the learned rule in the learning phase was consolidated in the test phase. The negative correlations in the incongruent supervised rule-learning conditions suggest that a switch from the learned rule in the learning phase to the utility-dominant option in the test phase. Because of the research design, which included the manipulation of utility in the test phase, mediation analysis to analyze the psychological mechanism of moral learning further, as in Experiment 1, was not possible. The lack of correlation in the unsupervised rule-learning conditions suggests that any learned rule in the learning phase was qualitatively different from and/or weaker than the rule learned in the supervised rule-learning conditions.

## General discussion

Evidence for the flexibility of moral preferences is more than a century old, with research recording human responses to hypothetical moral dilemmas dating back to Sharp (1898). However, despite a wealth of evidence for numerous contextual factors that induce moral flexibility, to the best of our knowledge, no experimental research has investigated moral flexibility in supervised moral rule-learning where novel rules are not associated with a typical moral strategy (e.g., utilitarianism) or culturally defined social expectation (e.g., queuing). We proposed and found that moral decision preferences are flexible and shift towards newly learned moral rules when their application leads to utilitarian choices.

In Experiment 1 we employed supervised and unsupervised rule-learning tasks where participants learned moral rules that corresponded to decision options in response to a moral scenario. Accordingly, consistent with *H1*, we demonstrated that within 30 learning trials participants learn new moral rules and transfer these rules to moral decision-making tasks where feedback is no longer provided. Crucially, rule 1 and rule 2 elicited approximately equal learning rates; therefore, rule-learning was not merely a result of participants favoring a particular set of stimuli under supervised reinforcement learning. Moreover, the results from Experiment 2 confirmed these findings. Specifically, we found evidence for positive transfer (congruent conditions) from the learned normative or dominant responses (learning phase of the study) to the responses in the test phase of the study. This happened when the utility-dominant option in the test phase was also congruent with the learned responses from the learning phase. Moreover, the results from the flexibility analysis revealed that with congruent rule-learning conditions the original effect size of positive transfer (Experiment 1) was increased by presenting a utility-dominant option in the test phase of Experiment 2. Therefore, these novel experiments have for the first time demonstrated that humans can learn entirely new moral rules through supervised and unsupervised learning task, and transfer to subsequent unsupervised decision-making.

Upon demonstrating that moral rules can indeed be learned through a supervised and unsupervised rule-learning tasks, we devised a second experiment to test *H2*, to establish whether decision-making based on supervised and unsupervised rule-learning alter in response to changes in the utility of decision options in the test phase of the experiment. Accordingly, the results from Experiment 2 revealed a shift in participants’ preferences; in incongruent conditions participants altered their initially learned responses (consistent with the learned rule) to the utility-dominant option in the test phase of the study. Moreover, the results from the flexibility analysis revealed that with incongruent rule-learning conditions the original effect size of positive transfer (Experiment 1) was reversed to negative by presenting an utility-dominant option in the test phase of Experiment 2. This result indicates induced negative transfer with an incongruent utility-dominant option available in the test phase of Experiment 2.

Participants who learned moral rules demonstrated a remarkably consistent decision preference for the learned rule when the utility-dominant options were congruent with the rule. To put the results of Experiment 2 into context, participants who learned moral rules, when subsequently presented with choices that had an incongruent utility-dominant option, shifted their decision-making strategy away from the learned rule to an incongruent utilitarian rule by saving the lives of a group of humans who were greater in number. Accordingly, in Experiment 2, moral flexibility through learning was demonstrated: humans learn rules, but subsequently shift their strategy to a rule that maximizes utility when one of the options dominates in terms of utility and the originally learned rule is incongruent with this dominance. Hence, when no utility dominant option exists, participants prefer to make choices that are consistent with recently learned moral rules. Participants accordingly follow a recency strategy. However, when there is a utility-dominant option available, and when this option is *not* consistent with the learned rule, participants switch from the recency strategy to a utilitarian strategy; they prefer the utility-maximizing option over the option consistent with the learned rule, further indicating moral flexibility. Thus, Experiment 2 reveals that in moral contexts, well established utilitarian strategy trumps recently learned moral rules. It is important to note here that the goal of our research was not to directly train participants to choose a non-utilitarian option. Doing so would entail implementing a utilitarian option in the learning phase, providing negative feedback each time the utilitarian option was chosen. It is plausible that following this method would result in active deviation away from utilitarian behavior in both the learning and transfer phase is since participants are directly trained *not* to make utilitarian choices. Our work is different. We do not actively train participants to deviate from utilitarian choice. Instead, in the learning phase of both Experiments 1 and 2, we presented novel moral rules in contexts where no utilitarian strategy is possible. In the transfer phase of Experiment 2 where no feedback is available we then introduced a utilitarian option which is either congruent or incongruent with the learned rule, to examine whether participants employ the learned strategy or the utilitarian strategy.

We utilized novel abstract rules to ensure learning occurred during and not before experimentation. In other words, participants had no prior experience with task stimuli or the rules we created. The drawback of this method is that such rules may not be representative of the real-world moral rules, which are often tangible and understood for their meaning (e.g., I will not litter because it harms the environment). However, the moral rules we have developed could be utilized in future experimental work. In our experimental studies, the moral scenario provided participants with the moral context for which they were making their moral choices. The rules themselves were abstract and could be utilized in moral and non-moral contexts to explore learning and transfer consistency. We speculate that the switch to utilitarian principles from learned rules may only occur in decision contexts with morally sensitive issues (i.e., the utility considered is human life as opposed to something not morally valuable; Kusev et al., 2016b). We invite future experimental work to investigate the extent to which our findings generalize to non-moral contexts.

However, our findings open new avenues for research in moral learning in naturalistic settings. In particular, future work could explore reinforcement learning involving rules and feedback that indicates social acceptance or majority opinion (Lindström et al., 2018) as well as rules tied to existing learned or innately prepared moral principles (Haidt & Joseph, 2004; Mikhail, 2007). Our research paradigm is based on abstract settings and calls for future work to explore reinforcement learning in real-world situations. Moreover, we also acknowledge here that our results do not suggest that moral flexibility can be explained by reinforcement learning alone. We speculate that the flexibility of moral preference is complex and there are circumstances where other forms of learning may outperform reinforcement learning. For example, Brady et al. (2021) reveal that when normative information is readily available, observation of moral norms can surpass reinforcement learning of expressed moral outrage. Hence, whilst reinforcement learning informs moral behavior, when social norms are available, they are a stronger predictor of moral behavior than reinforced feedback.

Since our experiments only involved tasks with corrective feedback or no feedback, we cannot rule out the possibility that learning could be achieved via mere contingency. It is possible that merely displaying the ‘correct’ response after the participant made each choice, with no corrective feedback, could result in participants consistently adopting a rule aligned with the ‘correct’ response (as they do with supervised learning). Kim and Hommel (2015) demonstrate that respondents’ judgements shift in the direction of responses that another agent has made, even when respondents are explicitly informed that the other agents’ responses were random. Hence, we do not rule out the possibility of learning via mere contingency in the absence of corrective feedback. Future studies could explore the effectiveness of mere contingency in this context and particularly determine whether learning consistency rates differ between mere contingency and supervised learning with corrective feedback.

Modern mediation analysis (Hayes, 2022) that we have conducted is common in psychological research, is well justified and is an advance over the traditional Baron and Kenny approach (Hayes, 2022). However, despite the random assignment to experimental conditions, we cannot guarantee unbiased estimates of causal mechanisms. As raised by Bullock et al. (2010), an exogenous independent variable does not extend to the mediator, which means it is possible that unmeasured confounders may still influence the mediator.

Participants in the unsupervised rule-learning condition demonstrated a preference for rule 2 across both Experiment 1 (in the learning phase and the test phase) and Experiment 2 (in the learning phase). Specifically, the option with the largest number of pips was chosen around 65% of the time. We offer two potential explanations for this. We speculate that this preference may be explained by participants attempting to find a way to maximize. Although, it was impossible for them to maximize lives saved in the learning phase, rule 2 was associated with a die with more pips and this may have been interpreted as a dominant option in the absence of any feedback.

The preference for rule 2 may alternatively be explained by participants’ response to their first impression of the stimuli. In particular, during the initial processing of the stimuli, it is plausible that due to the contrast of white pips on the blue die, the die with the larger number of pips was more visually salient that the die with a smaller number of pips. Accordingly, if no or reduced further processing takes place (i.e., evaluation of the content of each die), this initial visual salience could bias people’s attention to the die with the most pips, and influence participants’ decision-making preferences. Indeed, this explanation is consistent with empirical evidence which has demonstrated that visual salience can influence people’s risky and non-risky human judgment and decision-making (e.g., see Li & Camerer, 2022). However, the general preference for rule 2 amongst participants in the unsupervised rule-learning condition is of no consequence to our hypotheses. Moreover and crucially, this preference for rule 2 did not translate into an advantage in the learning phase or the test phase of the study under supervised rule-learning (as demonstrated in both experiments).

Overall, our empirical results demonstrate that moral learning is flexible (a normative or dominant response was formed within 30 learning trials), and it is plausible that our flexible moral system, by nature, learns and utilizes the most recent rules according to the environmental demands. Our work also advances theoretical accounts of moral reinforcement learning (e.g., Crockett, 2013; Gęsiarz & Crockett, 2015). Previous models have been developed to explain how humans learn to avoid aversive behavior (i.e., behaviors that cause harm to others), with a particular focus on deontological rules (Crockett, 2013). However, in our work we have not attempted to explore or induce utilitarian or deontological rule-learning. Instead, we have developed arbitrary rules with the purpose of establishing whether humans learn rules that are novel and disconnected from existing strategies, and then transfer these rules to contexts where feedback is no longer available.

## Conclusion

Our proposal of moral learning accounts for the use of a simple moral strategy which operates *on the fly* – where participants make future choices based on recent reinforcement histories indicating which behaviors are morally correct or incorrect. Our work has empirically revealed the flexible nature of moral preferences in response to rule-learning in the context of expected normative moral behavior. In light of our findings that people’s psychological concept of morality is continually under construction, future research should investigate how variation in moral-rule-learning (type of learned rules and quantity of simultaneously acquired rules) produces variations in moral behavior.

## Supplementary Information

Below is the link to the electronic supplementary material.

Supplementary file1 (PDF 157 KB)

## Data Availability

All data and R codes for Experiments 1 and 2 are available at OSF: https://osf.io/qj4dc/?view_only=4bb7bef36917411294c5bbc81315a80c.

## Footnotes

1 The lowest power was achieved for the independent t-tests: power = 0.86 for d = 0.8, power = 0.56 for d = 0.6, power = 0.22 for d = 0.4, power = 0.05 for d = 0.2.

2 The lowest power was achieved for the one-way ANOVA: power = 0.92 for f = 0.40, power = 0.43 for f = 0.25, power = 0.05 for f = 0.10.
