# Supplementary material for: Moral decision-making ‘on the fly’
Source: Psychol Res. 2025 May 8;89(3):98. doi: 10.1007/s00426-025-02126-z (PMC12062067; doi:10.1007/s00426-025-02126-z)
Supplement: Supplementary file 1 — (PDF 157 KB) [file 426_2025_2126_MOESM1_ESM.pdf]

Supplemental Materials

Moral decision making ‘on the fly’

by Kusev, P., Martin, R., van Schaik, P. & Teal, J.

Table S1

The presentation of each trial for the learning of Experiments 1 and 2 and test phase of Experiment 1. Each number denotes how many people were in each pip. For example, in. trial one (Learning Phase), ‘2’ indicates that there were two people inside one pip (Group A), and ‘1,1’ indicates that there were two people, each inside in separate pips (Group B).

Trial no.	Learning Phase		Test Phase	
	Group A	Group B	Group A	Group B
1	2	1,1	3	2,1
2	2,1	1,1,1	3,1	2,1,1
3	2,1,1	1,1,1,1	3,1,1	2,1,1,1
4	2,1,1,1	1,1,1,1,1	3,1,1,1	2,1,1,1,1
5	2,1,1,1,1	1,1,1,1,1,1	4	2,1,1
6	3	1,1,1	4,1	2,1,1,1
7	3,1	1,1,1,1	4,1,1	2,1,1,1,1
8	3,1,1	1,1,1,1,1	5	2,1,1,1
9	3,1,1,1	1,1,1,1,1,1	5,1	2,1,1,1,1
10	4	1,1,1,1	6	2,1,1,1,1
11	4,1	1,1,1,1,1	2,1,1,1,1	6
12	4,1,1	1,1,1,1,1,1	2,1,1,1	5
13	5	1,1,1,1,1	2,1,1,1,1	5,1
14	5,1	1,1,1,1,1,1	2,1,1	4
15	6	1,1,1,1,1,1	2,1,1,1	4,1
16	1,1,1,1,1,1	6	2,1,1,1,1	4,1,1
17	1,1,1,1,1	5	2,1	3
18	1,1,1,1,1,1	5,1	2,1,1	3,1
19	1,1,1,1	4	2,1,1,1	3,1,1
20	1,1,1,1,1	4,1	2,1,1,1,1	3,1,1,1
21	1,1,1,1,1,1	4,1,1		
22	1,1,1	3		
23	1,1,1,1	3,1		
24	1,1,1,1,1	3,1,1		
25	1,1,1,1,1,1	3,1,1,1		
26	1,1	2		
27	1,1,1	2,1		
28	1,1,1,1	2,1,1		
29	1,1,1,1,1	2,1,1,1		
30	1,1,1,1,1,1	2,1,1,1,1		

Table S2

The presentation of each trial for the two test phases of Experiment 2. Each number denotes how many people were in each pip. For example, in. trial one (Test Phase: Rule 1 is Utilitarian), '3' indicates that there were three people inside one pip (Group A), and '1,1' indicates that there were two people, each inside in separate pips (Group B).

Trial no.	Test Phase: Rule 1 is utilitarian		Test Phase: Rule 2 is utilitarian	
	Group A	Group B	Group A	Group B
1	3	1,1	2	1,1,1
2	3,1	1,1,1	2,1	1,1,1,1
3	3,1,1	1,1,1,1	2,1,1	1,1,1,1,1
4	3,1,1,1	1,1,1,1,1	2,1,1,1	1,1,1,1,1,1
5	4	1,1,1	3	1,1,1,1
6	4,1	1,1,1,1	3,1	1,1,1,1,1
7	4,1,1	1,1,1,1,1	3,1,1	1,1,1,1,1,1
8	5	1,1,1,1	4	1,1,1,1,1
9	5,1	1,1,1,1,1	4,1	1,1,1,1,1,1
10	6	1,1,1,1,1	5	1,1,1,1,1,1
11	1,1	3	1,1,1	2
12	1,1,1	3,1	1,1,1,1	2,1
13	1,1,1,1	3,1,1	1,1,1,1,1	2,1,1
14	1,1,1,1,1	3,1,1,1	1,1,1,1,1,1	2,1,1,1
15	1,1,1	4	1,1,1,1	3
16	1,1,1,1	4,1	1,1,1,1,1	3,1
17	1,1,1,1,1	4,1,1	1,1,1,1,1,1	3,1,1
18	1,1,1,1	5	1,1,1,1,1	4
19	1,1,1,1,1	5,1	1,1,1,1,1,1	4,1
20	1,1,1,1,1	6	1,1,1,1,1,1	5
